# Commentary: Supplier-dependent differences in intermittent voluntary alcohol intake and response to naltrexone in Wistar rats

**DOI:** 10.3389/fnins.2016.00082

**Published:** 2016-03-07

**Authors:** Allan V. Kalueff

**Affiliations:** ^1^Research Institute for Marine Drugs and Nutrition, College of Food Science and Technology, Guangdong Ocean UniversityZhanjiang, China; ^2^Institute of Translational Biomedicine, St. Petersburg State UniversitySt. Petersburg, Russia; ^3^Institutes of Chemical Technology and Natural Sciences, Ural Federal UniversityEkaterinburg, Russia; ^4^Neuroscience and Pharmacology Lab, ZENEREI InstituteSlidell, LA, USA

**Keywords:** behavior, phenomics, phenotypes, brain disorders, experimental models in neuroscience

Phenomics is an important area of biomedicine that focuses on how phenotypes are affected by various genetic and environmental factors (Houle et al., 2010). Because behaviors represent the most complex phenotypic traits crucial for organism adaptations and survival, *behavioral phenomics* is a new key field in translational neuroscience research (Gerlai, 2002). The impact of strain differences and procedural/experimental manipulations has received a considerable attention in the literature, including both behavioral and neuropsychopharmacological studies in mice and rats (McIlwain et al., 2001; Holmes et al., 2003a,b; Crabbe et al., 2008; Blokland et al., 2012).

Recently, Momeni et al. (2015) have reported supplier-dependent differences in alcohol intake, open field and Y-maze behavior, and responsivity to opioid antagonist naltrexone in outbred Wistar rats obtained from three different vendors in Europe. This timely and thorough study is important, as it further supports using genetically heterogeneous cohorts in preclinical behavioral research to develop experimental models with high population validity that better parallel human patients (Stewart and Kalueff, 2015).

However, several additional factors may be considered when interpreting the results of this study, performed in laboratory in Uppsala (Sweden) following a relatively short 2-week acclimation after obtaining animals from three foreign suppliers. Notably, the Harlan Laboratories at Horst (Netherlands) are located ~1500 km from Uppsala, compared to a 900-km distance to Taconic Farms at Ejby (Denmark) and nearly a 1700-km distance to the Charles River in Sulzfeld (Germany). The impact of transportation on psychological and physiological parameters in Wistar rats has recently been recognized (Arts et al., 2012, 2014a,b), raising the possibility that robust differences in transportation duration (~1700 vs. ~1500 vs. ~900 km) and, most likely, mode, packaging and shipment procedures, between the three suppliers could also contribute to overt behavioral differences reported by Momeni et al. (2015). Although usually this technical information is not described in-depth in neurobehavioral papers, the study of Momeni et al. (2015) suggests the opposite, indicating that vendor-related influences may indeed be critical in behavioral assays. Therefore, providing more detailed information on animal transportation from vendors to research laboratories (including distance traveled, mode of transportation (air/car), delivery procedures, and details of shipping/handling) can become best practices of neurobehavioral phenotyping. Eventually routinely reported in the Methods Sections of research papers, such details would be particularly important for replication of behavioral data, especially obtained in purchased (vs. raised in-house) animals and/or with short (e.g., 2–3 weeks) acclimation periods used.

Equally important here can be differences in animal housing between all four locations. For example, if rats' initial housing conditions in vendor A are similar to the testing site B, the animal transfer (and, respectively, acclimation) stress can be lesser than that caused by transfer from location C with distinct housing standards. Our analyses suggest that there may indeed be some of such site-specific differences. For example, the Charles River Sulzfeld facility houses up to seven Wistar rats 8 week old in open top Type IV cages (ground floor 1820 cm^2^) with Rettenmaier BK8/15 bedding and wooden gnawing sticks (personal communication, Research Models and Services, Charles River, Germany), compared to housing purchased animals 2–3 rats per a same-size cages with wood chip bedding and two 40 × 60-cm paper sheets as enrichment in Momeni et al. (2015). Thus, given a relatively short 2-week acclimation period used by Momeni et al. (2015), even subtle inter-facility differences in the number of animals per cage, cage type/color, rack type, ventilation, bedding, enrichment, and/or handling by experimenters all may affect the rats' responses following their transfer from vendor to the testing laboratory, regardless of the strain's source *per se*. Therefore, such housing information, often missing in behavioral papers using rodent models, can be critical for understanding strain differences and their modulation by various genetic and environmental modifiers.

Finally, analyzing several behavioral and physiological phenotypes in neurophenomics research can benefit from considering them as a system of potentially interplaying (rather than individual) phenotypes or their larger clusters, “domains” (Kalueff and Murphy, 2007; Kalueff et al., 2008; Stewart and Kalueff, 2014, 2015). For example, the overlap between anxiety and cognition has long been recognized in various rodent preclinical models (Kalueff and Murphy, 2007). Similar pathogenetic overlaps are also well-recognized for other phenotypic domains, including anxiety and addiction or social behaviors (Kalueff et al., 2008). Because such cross-phenotypic interplay mechanisms may reflect a true “integrative” nature of brain pathogenesis in question (Figure 1), their analyses can represent a useful strategy for building better and more valid neurobehavioral models (Kalueff and Murphy, 2007; Kalueff et al., 2008; Stewart and Kalueff, 2014, 2015). From this point of view, findings of Momeni et al. (2015), in our opinion, may generate several additional interesting insights into the pathogenic nature of their models. For example, applying Spearman correlation analysis to their summary subgroup data (Table 1 in Momeni et al., 2015) reveals a strong (–77%) negative correlation of rat sub-grouping in the open field (anxiety domain) and Y-maze performance (cognitive domain) between Harlan and Charles River Wistar rats, and a weaker correlation (–45%) for Harlan vs. Taconic Farms' rats. In contrast, correlation between the open field and alcohol intake (addiction domain) summary sub-grouping was strongly negative (–67%) for Harlan vs. Charles River, but positive and weak (17%) for Harlan vs. Taconic Farms' Wistar rats. Albeit more accurate results may be generated from correlating actual behavioral activity levels (rather than cohort sub-grouping) between various tests and vendors, this crude analysis suggests the possibility of strain-specific differences in the higher-order phenotypes, such as the extent of overlap between anxiety-like behavioral performance, cognitive functions, ethanol intake and sensitivity to naltrexone. Given the growing evidence of clinical comorbidity between affective, cognitive and drug abuse-related disorders (see Momeni et al., 2015 for details), further studies of strain- and vendor-specific inter-phenotypic correlations or overlaps of rat behavioral and pharmacological responses may be particularly valuable.

**Figure 1:**
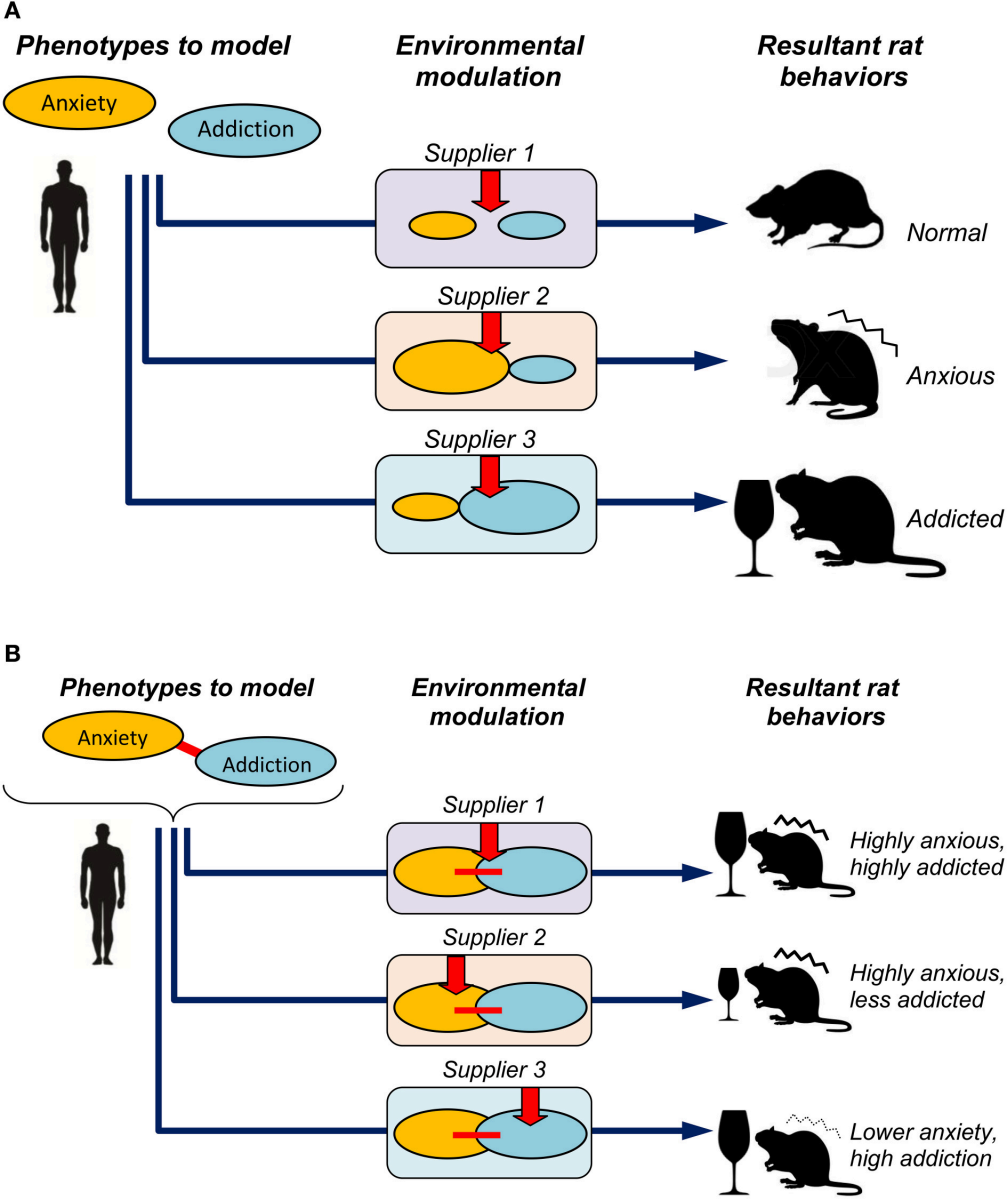
**Potential factors focusing on individual phenotypes (A) and additional factors modeling their cross-phenotypic interplay (B) in developing valid models of brain deficits**. In panel **(A)**, three different suppliers may differentially modulate the two examined phenotypes (e.g., anxiety and addiction), thereby resulting in distinct, supplier-specific resultant phenotypes in the same (Wistar) rat strain, similar to the modulation reported in Momeni et al. (2015). In panel **(B)**, the two core disordered phenotypes in question (denoted by ovals) are linked into a system of pathogenetic interplay (denoted by a red connecting line), to reflect a “true,” clinically relevant and well-defined disorder (e.g., mimicking common addiction problems in clinical anxiety). Respectively, in addition to supplier-specific modulation shown in panel **(A)**, the suppliers can differentially affect the neural mechanisms linking the two phenotypes into a system of common pathogenesis, thereby resulting in further phenotypic variance (i.e., high anxiety + high addiction, high anxiety + lower addiction, and lower addiction + higher anxiety) in the same rat strain.

## Author contributions

The author confirms being the sole contributor of this work and approved it for publication.

### Conflict of interest statement

The author declares that the research was conducted in the absence of any commercial or financial relationships that could be construed as a potential conflict of interest.
